# The Epigenomic Features and Potential Functions of PEG- and PDS-Favorable DNA G-Quadruplexes in Rice

**DOI:** 10.3390/ijms25010634

**Published:** 2024-01-04

**Authors:** Ranran Huang, Yilong Feng, Zhicheng Gao, Asgar Ahmed, Wenli Zhang

**Affiliations:** State Key Laboratory of Crop Genetics & Germplasm Enhancement and Utilization, CIC-MCP, Nanjing Agricultural University, No.1 Weigang, Nanjing 210095, China; 2021101068@stu.njau.edu.cn (R.H.); 2017201057@njau.edu.cn (Y.F.); 2022101019@stu.njau.edu.cn (Z.G.); asgar@stu.njau.edu.cn (A.A.)

**Keywords:** G4s, PEG, PDS, epigenetic features, functions, rice

## Abstract

A G-quadruplex (G4) is a typical non-B DNA structure and involved in various DNA-templated events in eukaryotic genomes. PEG and PDS chemicals have been widely applied for promoting the folding of in vivo or in vitro G4s. However, how PEG and PDS preferentially affect a subset of G4 formation genome-wide is still largely unknown. We here conducted a BG4-based IP-seq in vitro under K^+^+PEG or K^+^+PDS conditions in the rice genome. We found that PEG-favored IP-G4s^+^ have distinct sequence features, distinct genomic distributions and distinct associations with TEGs, non-TEGs and subtypes of TEs compared to PDS-favored ones. Strikingly, PEG-specific IP-G4s^+^ are associated with euchromatin with less enrichment levels of DNA methylation but with more enriched active histone marks, while PDS-specific IP-G4s^+^ are associated with heterochromatin with higher enrichment levels of DNA methylation and repressive marks. Moreover, we found that genes with PEG-specific IP-G4s^+^ are more expressed than those with PDS-specific IP-G4s^+^, suggesting that PEG/PDS-specific IP-G4s^+^ alone or coordinating with epigenetic marks are involved in the regulation of the differential expression of related genes, therefore functioning in distinct biological processes. Thus, our study provides new insights into differential impacts of PEG and PDS on G4 formation, thereby advancing our understanding of G4 biology.

## 1. Introduction

A G-quadruplex (G4) is a type of non-B-type DNA secondary structure containing four planar structures with guanine-rich nucleic acid sequences, which are stabilized via Hoogsteen-type hydrogen bonding [1]. G4s were initially found to be spontaneously formed with high concentrations of guanine in 1910 [2], but their real structures were first discovered in guanylate (GMP) gels through X-ray diffraction [3], which acted as a starting point to boost the in silico and experimental studies of G4s across various prokaryotic and eukaryotic genomes, including *E. coli*, yeast, humans and plants [4,5,6,7,8,9]. The genome-wide prediction of putative G-quadruplex forming sequences (PQFSs) shows that G4s exhibit a genomic-region-dependent distribution [10,11,12,13], such as an overrepresentation in promoters in bacteria, yeast and humans [4,5]. In agreement with their genomic distributions, G4s have been found to be involved in various DNA-templated events, including DNA replication [14], telomere activities [15,16], transcriptional and translational regulations [17,18], epigenetic regulations [19], chromatin remodeling [20,21], an increase of species-related evolutionary complexity through coevolution with mitochondria [22], and human diseases [23,24] as well as stress responses in both animals and plants [6,25,26,27]. In particular, G4s have been emerging as promising targets for drug design [28], and the diagnosis and treatment of human diseases [23,24]. Therefore, G4s have become a hot research topic in humans.

The availability of high-throughput sequencing-based methodologies has greatly advanced G4 biology in various eukaryotic genomes, including mice, humans and plants. They include anti-BG4-based ChIP-seq in humans [29] and DNA IP-seq in rice [7], D1 antibodies-based ChIP-seq in humans [30,31], G4-seq in multiple species including *Arabidopsis* [32], an artificial truncated DHX36 helicase-based G4P-ChIP in human, mouse, and chicken cells [33], CUT&Tag (cleavage under targets and tagmentation) in mammalian cells [34], and BioTASQ- and BiocyTASQ-based G4DP-seq in rice [35].

Growing evidence demonstrates that in vitro G4 formation and stabilization can be affected by intrinsic DNA sequences and external environmental factors, including monovalent cations such as Na^+^ and K^+^, in particular, K^+^ can stabilize G4 structures by counteracting the electrostatic repulsion of the negatively charged C6 oxygen atoms [36,37]; molecular crowding, which mimics the cellular environment to favor G4 formation or stabilization [38,39]—for instance, polyethylene glycol (PEG), has been reported to promote in vitro G4 formation [40,41,42,43]; small-molecule G4 ligands such as pyridostatin (PDS) and its derivatives with a high specificity for the binding of G4s [44], which have been applied to favor a subset of G4 formation genome-wide [45] and to visualize G4 structures in vitro [46] and in living cells [47]. Furthermore, PDS can protect the 5′-tetrad of the G4 structure against oxidation, thereby impeding its reconfiguration into a double helix [48].

Compared to the fruitful progress of G4s in non-plant systems, G4 studies in plants are still at the early stage, even though the global prediction of PQFSs has already started in several plant species [8,25,49,50,51,52,53,54], including *Arabidopsis*, rice, maize, wheat and barley, and genome-wide experimental characterizations of G4s have been initiated in *Arabidopsis* [32] and rice [7,35,55]. G4 studies in plants indicate that plant G4s can be involved in various biological processes, including the regulation of gene expression and translation, TE activities, telomere functions, epigenetic modifications, and plant growth and development, as well as stress responses [6,7,8,25,27,53,56,57,58,59]. More efforts still need to be invested to elucidate the functions of G4s in plants.

PEG and PDS chemicals have already been applied to promote G4 folding in vitro in rice [7,35,55] and *Arabidopsis* [32], respectively. However, how PEG and PDS preferentially affect a subset of G4 formation genome-wide is still largely unknown. In this study, we conducted BG4-based IP-seq under K^+^+PEG or K^+^+PDS conditions for the identification of PEG- or PDS-favorable in vitro G4s, labeled IP-G4s^+^, representing BG4-captured G4s in vitro, followed by genetic, epigenetic and functional characterizations of both subtypes of G4s in the rice genome.

## 2. Results

### 2.1. Identification of PDS/PEG-Specific IP-G4s^+^

**Figure 1:**
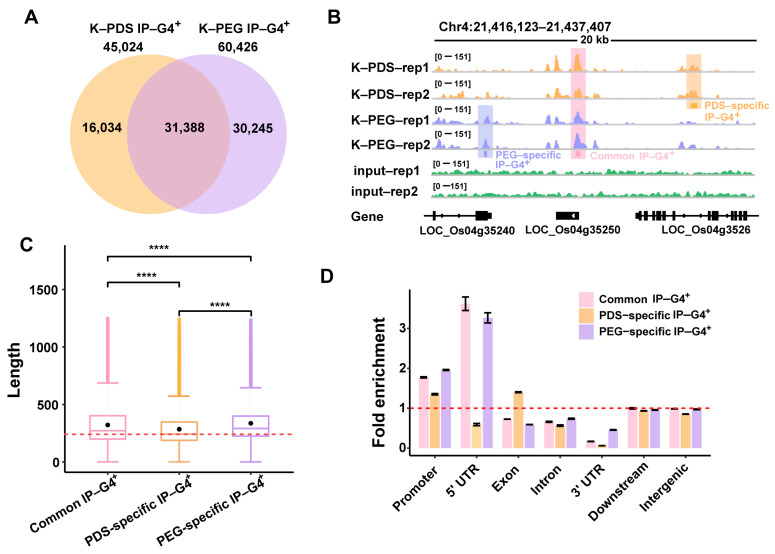
Identification of common and PDS/PEG-specific IP-G4s. (**A**) Venn diagram illustrating the number of the three subtypes of IP-G4s peaks (PEG-specific, PDS-specific, common ones). (**B**) A representative snapshot of Integrative Genomics Viewer (IGV) spanning a 20 kb window illustrating distributions of common and PEG/PDS-specific IP-G4 peaks in the rice genome. Each colored box represents a distinct subtype of IP-G4 peaks. (**C**) Boxplot showing the length of different subtypes of IP-G4 peaks. Significance test was performed using the Wilcoxon rank-sum test. **** *p*-value < 0.0001. (**D**) Distributions of observed relative to expected IP-G4s within each sub-genomic region in the rice genome. A ratio of 1.0, represented by a red dashed line, indicates an equal distribution.

It has been reported that PDS or PEG helps stabilize G4 structures, thereby facilitating G4 formation [60]. However, the differential impacts of PDS and PEG on G4 formation on a genome-wide scale are still largely unknown. To address this question, we performed a BG4-DNA-IP-seq as described previously [55] for the identification of in vitro G4s under K+PEG or PDS conditions. We obtained two well-correlated biological replicates for each condition (R = 0.90 and 0.87 for K+PEG/PDS, respectively) (Figure S1; Table S1). We then identified 60,426/45,024 biologically reproducible IP-G4^+^ peaks under K+PEG/PDS conditions, respectively (Figure S2), leading to 16,034/30,245 PDS- and PEG-specific IP-G4s^+^, respectively, and 31,388 common ones (Figure 1A). A representative Integrative Genomics Viewer (IGV) spanning a 20 kb region from the rice Chromosome 4 shows the reproducible peaks of common and PEG/PDS-specific IP-G4s^+^ between replicates (Figure 1B). We then randomly selected three loci of PEG- or PDS-specific IP-G4s^+^ for a circular dichroism (CD) spectroscopy assay. We observed that each oligo from PEG-specific or PDS-specific IP-G4^+^ exhibited a higher CD absorption peak when the oligo was reconstructed in buffer containing PEG or PDS compared to the same oligo reconstructed in a buffer containing PDS or PEG, respectively (Figure S3). After overlapping with 1,797,039 putative G-quadruplex-forming sequences (PQFSs) predicted using the fastaRegexFinder.py script [61] as previously described [8], we found that approximately 94.8% of PEG- and 93.5% of PDS-related IP-G4s^+^ peaks contain PQFSs (Figure S4).

K^+^- and Na^+^-favorable IP-G4^+^ peaks have length variations [55], inspiring us to look into the length of PEG/PDS-specific IP-G4s^+^. we found that PEG-specific IP-G4s had the longest mean length (~330 bp), while PDS-specific ones had the shortest mean length (~290 bp), indicative of the length variations of IP-G4s^+^ between PDS and PEG conditions (Figure 1C). We then examined the genomic distributions of the three subtypes of IP-G4s^+^ and observed that PEG-specific IP-G4s^+^ were more distributed in promoters, introns and 3′UTRs, but less distributed in intergenic and downstream regions, and in exons, compared to PDS-specific ones (Figure S5). According to the fold enrichment of observed to expected structures, we found that PEG-specific IP-G4s^+^ were more enriched in 5′UTRs, while PDS-specific IP-G4s^+^ were more enriched in exons. For promoters with all three subtypes of IP-G4^+^ peaks, PDS-specific IP-G4s^+^ had the lowest enrichment levels compared to the other two subtypes of IP-G4s^+^ (Figure 1D).

### 2.2. Genomic Features of Each Subtype of IP-G4s^+^

**Figure 2:**
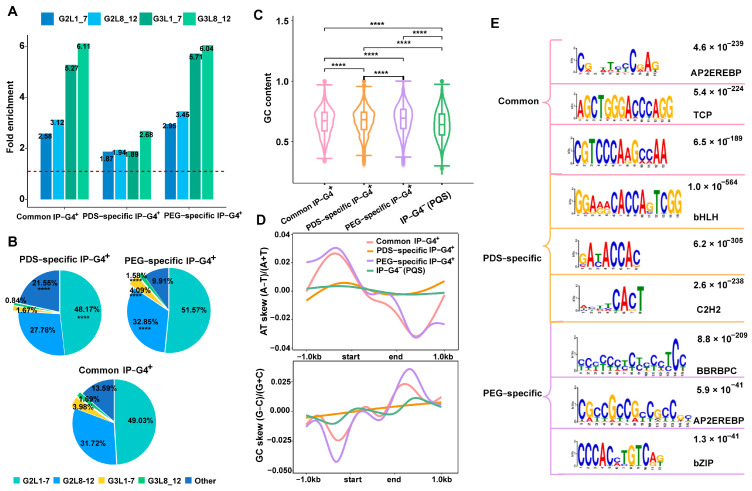
Sequence features of each subtype of IP-G4s. (**A**) Fold enrichment of each subtype of PQFSs, as indicated, in PDS/PEG-specific and common IP-G4s. (**B**) The percentage of each subtype of PQFSs, as indicated, in PDS/PEG-specific and common IP-G4s. (**C**) GC content of PDS/PEG-specific and common IP-G4s and IP-G4s^−^ (PQFSs). Significance test was performed using the Wilcoxon rank-sum test. **** *p*-value < 0.0001. (**D**) GC and AT skews of PDS/PEG-specific and common IP-G4s (PQFSs) in both forward and reverse DNA strands calculated at approximately ±1 kb. (**E**) De novo motif identification for common and PEG/PDS-specific IP-G4 peaks using MEME. The top three significantly enriched motifs are listed.

To assess the sequence features of the three subtypes of IP-G4s^+^, we calculated the number of four typical subtypes of PQFSs, including G2L1–7, G2L8–12, G3L1–7 and G3L8–12. We found that each subtype of IP-G4s^+^ had a similar trend of subtypes of PQFSs with the descending order G2L1–7, G2L8–12, G3L1–7 and G3L8–12 (Figure S6), which is consistent with our previous findings [55]. We then calculated the fold enrichment of each subtype of PQFSs within each subtype of the IP-G4s^+^ relative to randomly selected genomic regions with the same number and size as each subtype of the IP-G4s^+^. We observed that PEG-specific and common IP-G4s^+^ had higher enrichment levels of G3L1–7 and G3L8–12, while PDS-specific IP-G4s^+^ had the lowest enrichment levels of each subtype of PQFSs (Figure 2A). Except for non-typical types of PQFSs, PDS-specific IP-G4s^+^ had significantly lower levels of all the subtypes of typical PQFSs compared to PEG-specific ones (Figure 2B). Moreover, the genomic distributions of subtypes of PQFSs exhibited a subtle difference within each subtype of IP-G4s^+^ (Figure S7). For instance, PDS-specific IP-G4s^+^ had the smallest percentage of each subtype of PQFSs distributed in 5′UTRs, and had more G2L1–7, G2L8–12 and G3L8–12 distributed in exons.

After examining the GC content and GC/AT skew, we found that the GC content was exhibited in a descending order from PEG-specific to PDS-specific and common IP-G4s+, and then IP-G4s^−^ (PQFSs) (Figure 2C). For GC/AT skew, we found that PEG-specific and common IP-G4s^+^ (PQFSs) exhibited more pronounced GC/AT skew than the other two subtypes of G4s; in sharp contrast, almost no GC/AT skew occurred for PDS-specific IP-G4s^+^ (PQFSs) (Figure 2D). To interrogate whether PDS- and PEG-specific IP-G4s^+^ have distinct biological implications, we conducted de novo motif identification for common and PDS/PEG-specific IP-G4s^+^. According to the top three most significantly enriched motifs, we found that the motifs for the binding of bHLH and C2H2 transcription factors (TFs) were more enriched in PDS-specific IP-G4s^+^; in contrast, the motifs for the binding of BBRBPC and bZIP TFs were more enriched in PEG-specific IP-G4s^+^ (Figure 2E). These TFs have been reported to play crucial roles in the regulation of plant growth and development and stress responses [62,63,64,65,66].

Collectively, these results indicate that PDS- and PEG-specific IP-G4s^+^ have distinct sequence features and biological functions, including the regulation of TEGs and non-TEGs.

### 2.3. Distinct Associations of PDS/PEG-Specific IP-G4s^+^ with TEs or TEGs

**Figure 3:**
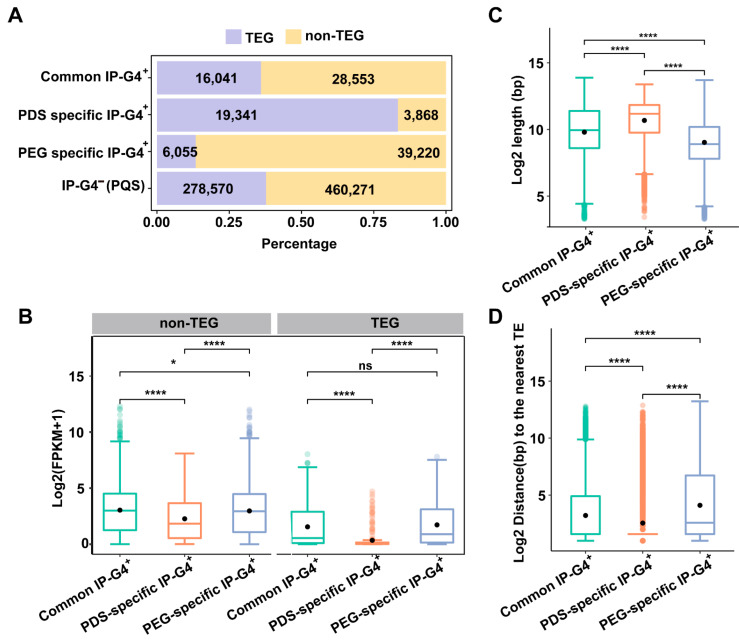
Characterization of PDS/PEG-specific IP-G4s^+^ associated with TEGs and TEs. (**A**) Distributions of PDS/PEG-specific and common IP-G4s^+^ (PQFSs), and IP-G4s^−^ (PQFSs), within TEGs and non-TEGs. (**B**) Expression levels of non-TEGs and TEGs associated with each subtype of IP-G4s^+^, as indicated. (**C**) The length of TEs associated with each subtype of IP-G4s^+^, as indicated. (**D**) The distance of TEs associated with each subtype of IP-G4s^+^, as indicated, to the nearest TEs. Significance test was performed using the Wilcoxon rank-sum test. * *p*-value < 0.05, **** *p*-value < 0.0001, ns nonsignificant difference.

G4s have been proposed to be involved in the regulation of the transposon life cycle [8]. To investigate distributions of PDS/PEG-specific IP-G4s^+^ within TEs or TEGs, we associated them with transposable element genes (TEGs) and non-transposable element genes (non-TEGs), and found that over 80% of PDS-specific IP-G4s^+^ (PQFSs) were associated with TEGs, while more than 80% of PEG-specific IP-G4s^+^ (PQFSs) were associated with non-TEGs (Figure 3A). We then calculated the expression levels of the TEGs and non-TEGs associated with each subtype of IP-G4s^+^, and found that non-TEGs associated with PDS-specific IP-G4s^+^ had the lowest mean expression levels, while those associated with PEG-specific IP-G4s^+^ had the highest mean expression levels, and a similar expression trend occurred for the TEGs associated with PDS/PEG-specific IP-G4s^+^ (Figure 3B).

We then looked into the distributions of each subtype of IP-G4s^+^ within Class I (Retrotransposons) and II (DNA transposons) TEs, and found that PDS-specific IP-G4s^+^ (PQFSs) were overrepresented (78%) in Class I TEs, while PEG-specific IP-G4s^+^ (PQFSs) were more prevalently distributed (77%) in Class II TEs (Figure S8). After calculating the length of the TEs associated with each subtype of IP-G4s^+^ (PQFSs), we found that the TEs associated with PDS-specific IP-G4s^+^ (PQFSs) had the longest length; in contrast, the TEs associated with PEG-specific IP-G4s^+^ (PQFSs) had the shortest length (Figure 3C). A similar trend was observed for the length of the Class I and II TEs associated with each subtype of IP-G4s^+^ (PQFSs) and the length of the Class II TEs was shorter compared to Class I TEs with their corresponding IP-G4s^+^ (PQFSs) (Figure S9). To examine the density of the TEs associated with each subtype of IP-G4s^+^ (PQFSs), we calculated the distance of the TEs associated with each subtype of IP-G4^+^ to the nearest TEs, and found that TEs associated with PDS-specific IP-G4s^+^ (PQFSs) had the shortest distance, while those associated with PEG-specific IP-G4s^+^ (PQFSs) had the longest distance (Figure 3D). Again, a similar trend was observed for the Class I and II TEs associated with each subtype of IP-G4s^+^ (PQFSs) and the distance of Class II TEs to the nearest TEs was much longer than that of Class I TEs with their corresponding IP-G4s^+^ (PQFSs) (Figure S10). These results suggest that PDS- and PEG-specific IP-G4s^+^ may have distinct roles in the regulation of TEGs and non-TEGs and the activities of Class I and Class II TEs.

### 2.4. Epigenomic Features of PEG/PDS-Specific and Common IP-G4s^+^

**Figure 4:**
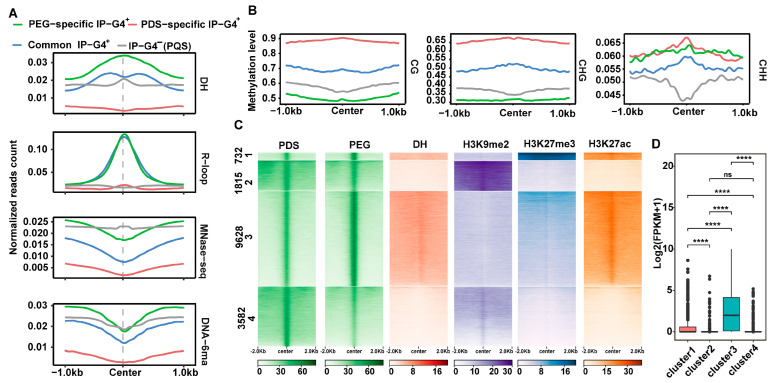
Epigenomic features of PEG/PDS-specific and common IP-G4s^+^. (**A**) Normalized read counts of DNase-seq (DHSs), MNase-seq (nucleosomes), DRIP-seq (R-loops) and DNA-6mA-IP-seq (6mA) were plotted across ±1 kb of the center of PEG/PDS-specific and common IP-G4s^+^ (PQFSs) and IP-G4s^−^ (PQFSs). (**B**) CG, CHG and CHH methylation levels were calculated across ±1 kb of the center of PEG/PDS-specific and common IP-G4s^+^ (PQFSs) and IP-G4s^−^ (PQFSs) with similar C contents. (**C**) Heat maps showing the results of K-mean clustering for PDS- or PEG-related IP-G4s, DHSs, histone marks (H3K9me2, H3K27me3 and H3K27ac). (**D**) The boxplot illustrating the mean expression levels of genes associated with each subcluster identified in (**C**). Significance test was performed using the Wilcoxon rank-sum test. **** *p*-value < 0.0001, ns *p*-value > 0.05.

To explore the epigenomic features of PDS- or PEG-specific IP-G4s^+^ (PQFSs), we plotted normalized read counts of DNase-seq (DHSs), DRIP-seq (R-loops), MNase-seq (nucleosomes) and DNA-6mA IP-seq across ±1 kb of the midpoint of each subtype of IP-G4s^+^ (PQFSs) and IP-G4s^−^ (PQFSs). We found that PEG-specific IP-G4s^+^ exhibited the highest enrichment levels of DHSs and R-loops across all regions examined, compared to the other subtypes of IP-G4s^+^, but less enrichment levels of their nucleosomes and DNA-6mA at around the midpoint, compared to IP-G4s^−^ (PQFSs); in contrast, PDS-specific IP-G4s^+^ exhibited the lowest enrichment levels of the four epigenomic marks tested across all regions (Figure 4A). We then calculated the DNA methylation levels in each cytosine context at around ±1 kb of the midpoint of each subtype of IP-G4s^+^ (PQFSs) and IP-G4s^−^ (PQFSs). We found that PDS- and PEG-specific IP-G4s^+^ had the highest and lowest levels of CG and CHG methylation, respectively. Moreover, PDS- and PEG-specific IP-G4s^+^ had higher CHH methylation levels than the other two subtypes of G4s (Figure 4B). We finally conducted clustering analyses for PEG/PDS-specific IP-G4s^+^ in combination with DHSs, H3K9me2, H3K27me3 and H3K27ac marks, and divided all PEG/PDS-specific IP-G4s^+^ into four subclusters with distinct epigenomic features (Figure 4C). For instance, subcluster 1 exhibited high enrichment levels of DHSs, H3K27me3 and H3K27ac, a combination of active and repressive marks; subclusters 2 and 4 were mainly co-localized with the repressive mark, H3K9me2; and subcluster 3 exhibited high enrichment levels of the active marks, DHSs and H3K27ac. Accordingly, after comparing the expression levels of the genes associated with each subcluster, we found that the genes in subcluster 3 had the highest expression levels, genes in subcluster 1 had the second highest expression levels, and genes in subclusters 2 and 4 had lower expression levels compared to those in the other two subclusters (Figure 4D). We found that TEGs with PEG-specific IP-G4s^+^ had the highest enrichment levels of DNA-6mA and that non-TEGs with PDS-specific IP-G4s^+^ had the lowest enrichment levels of DNA-6mA (Figure S11). This is consistent with the results we previously observed for PEG/PDS-specific IP-G4s^+^.

Taken together, these results show that PEG-specific IP-G4s^+^ tend to be associated with euchromatin, while PDS-specific IP-G4s^+^ tend to be associated with heterochromatin. PEG/PDS-specific IP-G4s^+^ may coordinate with epigenetic marks to affect the expression of overlapping genes.

### 2.5. Relationships of PEG/PDS-Specific IP-G4s^+^ with the Expression Levels of Overlapping Genes

**Figure 5:**
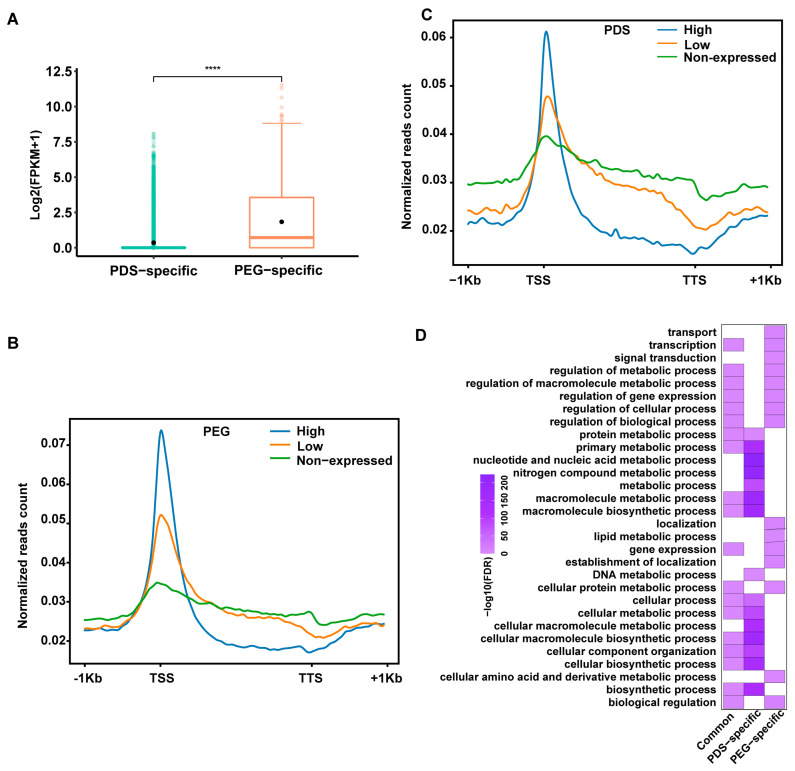
Relationships of PEG/PDS-specific IP-G4s^+^ with expression of overlapping genes. (**A**): Boxplots showing expression levels of genes associated with PEG- and PDS-specific IP-G4s^+^. (**B**,**C**): curve plots showing distributions of normalized read counts of PDS- and PEG-related IP-G4s^+^ across ±1 kb of the TSSs of overlapping genes with different expression levels (high, low and no expression, FPKM values). (**D**) GO terms enrichment assays for genes associated with PEG/PDS-specific and common IP-G4s^+^ (PQFSs). Significance test was performed using the Wilcoxon rank-sum test. **** *p*-value < 0.0001.

It has been documented that G4s exhibit genomic position-dependent impacts on the expression of associated genes [55]. To assess the relationships of PEG/PDS-specific IP-G4s^+^ with the expression of overlapping genes, we compared the expression levels of genes associated with PDS/PEG-specific IP-G4s^+^, and found that genes with PEG-specific IP-G4s^+^ had higher expression levels than genes with PDS-specific IP-G4s^+^ (Figure 5A). We then plotted normalized read counts of PDS/PEG-specific IP-G4s^+^ across ±2 kb from the TSSs to the TTSs of genes with different expression levels (high, low and no expression, FPKM values). In agreement with our previous findings [67], we found that PDS/PEG-specific IP-G4s^+^ in promoters and gene bodies exhibited a positive and negative association with the expression levels of associated genes, respectively (Figure 5B,C). According to GO enrichment analyses, we found that genes with PDS-specific IP-G4s^+^ had distinct GO terms compared to those with PEG-specific IP-G4s^+^ (Figure 5D). For instance, genes with PDS-specific IP-G4s^+^ had GO terms related to nucleotide/DNA/cellular macromolecule/nitrogen compound metabolic processes; in contrast, PEG-specific IP-G4s^+^ had GO terms with functions associated with transport, signal transduction, lipid/cellular amino acid and derivative metabolic processes, and localization. These results show that PEG/PDS-specific IP-G4s^+^ have differential impacts on the expression of related genes, which have distinct biological implications.

## 3. Discussion

It has been well documented that plenty of internal and external factors affect G4 formation or stability in vitro [68,69], their combined actions influence the topological changes in parallel and antiparallel G4s [70]. Internal factors include the intrinsic sequence composition such as the number and length of G-tracts [71,72], the size, position and sequence composition of loops [68,72,73,74,75], the flanking base [76], DNA chemical modifications such as 5mC [77], O6-Methylguanine [78], 8-Aminoguanine [79], 8-Methylguanine [80], 8-bromoguanine [81] and inosine [82] and chromatin modifications [77]; external factors include monovalent cations [83,84,85], PEG-mimicked molecular crowding [41,86,87], pH [88], temperature [89], other additive agents such as polyamines [90] and trithylene tetraamine [91], and small ligands for the binding of G4s such as pyridostatin (PDS) [92], RHPS4 [93] and MM41 [94]. However, detailed investigations of the differential impacts of PEG and PDS on G4 formation on a genome-wide scale are still less studied. Our study showed that PEG favored greater G4 formation than PDS, indicative of the differential impacts of both chemicals on G4 formation. As mentioned above, this could be partly contributed to by distinct the genetic and epigenetic features between both subtypes of G4s (PQFSs), including the presence of distinct G4 sizes, a higher GC content, a distinct percentage of typical PQFSs such as G3L1–12, subgenomic distributions and epigenetic features such as DHSs, R-loops, DNA-6mA, DNA-5mC and histone marks. It has been reported that PEG and PDS have distinct mechanisms underlying G4 formation/stability or its topological changes [69,87]. Molecular crowding conditions favor the aggregation of G4s with parallel structures, partly contributed to by hydration-mediated thermodynamics [86,95,96], or its hydrophobic nature-related interactions with G4s [97,98], or its affect on G4 folding potentials [96,99]. PEG exhibits a Na^+^- or K^+^-dependent effect on the transition of G4s from a hybrid to a parallel structure [86,100]. For instance, G3 PQFSs tend to form a parallel structure under K^+^+PEG conditions [101]. PDS, as a lead G4-stabilizing ligand, has been widely applied for the local and global detection of G4s in several species due to its ability to bind and stabilize G4 motifs [32,46,92,102]. Interactions between PDS and G4 are partly determined by G4 sequences, topologies and chemical groups [103], thereby preferentially affecting distinct DNA-templated biological processes [46,92,104].

Furthermore, our study showed that PEG-favored G4s primarily associated with non-TEGs and Class II TEs, which were primarily located in euchromatic regions with active marks and lower DNA methylation levels, while PDS-favored G4s associated with TEGs and Class I TEs, which were preferentially enriched in heterochromatic regions with repressive marks and higher DNA methylation levels. It has been documented that transient and persistent G4s have distinct impacts on DNA methylation [21]. Promoter G4s of *cMYC*, *MEST* and *CDKN1C* exhibited a binding affinity in vitro to the de novo DNA methyltransferases DNMT3A and 3B, and the DNA methyltransferase DNMT1 as well [105], indicating that these G4s may play key roles in establishing or maintaining local DNA methylation. In sharp contrast, a subset of G4s have been found to sequester DNMT1 accessibility, thereby contributing to local DNA hypomethylation [19]. These contrasting findings suggest that the effects of G4s on local DNA methylation changes may be G4 conformation- and genomic location-dependent. DNA methylation has been found to stabilize the promoter G4s through inducing conformational changes [106,107]. In addition, DNA or RNA G4s have been found to interact with histone chaperones, histone modifiers and chromatin remodelers to affect chromatin dynamics such as histone modifications, nucleosome density and chromatin compactness [21,108]. For instance, it has been reported that the binding partners of the promoter G4 of *c-Myc* contain a subunit of SWI/SNF and NuRD, and subunits of the PRC2 [21]. Similarly, our study also suggests that different G4 conformations in different genomic regions may have distinct associations with epigenetic marks. Accordingly, genes associated with PEG-specific G4s had higher expression levels than those associated with PDS-specific G4s, suggesting that these two subtypes of G4s alone, or coordinating with distinct chromatin marks, affect the differential expression of related genes, thereby being involved in distinct biological processes.

Thus, this study for the first time provides new insights into the differential impacts of PEG and PDS on G4 formation on a genome-wide scale, thereby advancing our understanding of G4 biology.

## 4. Materials and Methods

### 4.1. Growth of Rice Seedlings

The rice seeds (*Oryza sativa* L.) of cultivar Nipponbare (Japonica) were pregerminated in tap water at room temperature (RT) for 3 days. After being evenly spread onto a surface of nutrient soil in a tray and covered with a matched plastic cover to avoid water evaporation, uniformly germinated rice seeds were grown in a greenhouse with growth conditions of 28–30 °C and a 14 h/10 h light/dark cycle. Biological replicates of two-week-old rice seedlings above ground were collected for cross-linking. Cross-linking was conducted with a 1% final concentration of formaldehyde in HEPES buffer pH = 8.0 (20 mM HEPES, 1 mM EDTA, 100 mM NaCl and 1 mM PMSF) at 23–25 °C for 10 min under vacuum. A final concentration of 0.125 M glycine was used to completely quench the excessive formaldehyde under vacuum for an additional 5 min. The cross-linked seedlings were ground into fine powder in liquid nitrogen for the preparation of genomic DNA for downstream IP-seq assays.

### 4.2. BG4-DNA-IP-Seq

DNA immunoprecipitation with the anti-BG4 antibody coupled with sequencing, called BG4-DNA-IP-seq, was performed exactly according to our previously published protocols [7]. Briefly, 5.0 μg of fragmented genomic DNA was diluted in G4-stabilizing buffer (150 mM KCl and 10 mM Tris-HCl, pH = 7.5), then denatured and reassociated by letting the temperature slowly to drop down to RT. The re-associated DNA was diluted with G4-IP incubation buffer (50 mM HEPES, 150 mM KCl, 1 mM MgCl_2_, 130 nM CaCl_2_, 1% BSA (*w/v*), 5.0 μM PDS (or 40% PEG), Complete mini, pH = 7.5), which was then followed by incubation with 4.0 μg of anti-BG4-FLAG antibody in the IP incubation buffer for 4 h. After adding 3.0 μg of anti-FLAG antibody (D110005, BBI, Shanghai, China) for incubation for an additional 4 h at 4 °C, 30 μL of washed protein G Dynalbeads (10004D, Invitrogen, Carlsbad, CA, USA) was added for incubation for an additional 4 h, followed by washing two times. BG4-bound DNA was eluted with 200 μL elution buffer (0.1 M NaHCO_3_ and 1% SDS *w/v*) at 65 °C two times, for 15 min each. Anti-BG4-recognized DNA-G4s were finally recovered for library preparation.

For BG4-DNA-IP-seq library preparation and sequencing, two biologically replicated BG4-IPed DNAs and one replicate of Input/IgG-/anti-FLAG only IPed DNA (control) were used for library preparation using the NEBNext^®^ Ultra™ II DNA Library Prep Kit for Illumina (NEB, E7645S). All prepared libraries were sequenced on the Illumina platform with 150 bp paired-end mode, which was conducted by the company Berrygenomics (Beijing, China).

### 4.3. Analyses of BG4-DNA-IP-Seq Data

Low-quality reads were removed from raw data using Trim Galore! (Version 0.4.4, Felix Krueger, Cambridge, UK). All cleaned reads were aligned to the reference genome MSU v7.0. (http://rice.plantbiology.msu.edu/pub/data/Eukaryotic_Projects/o_sativa/annotation_dbs/pseudomolecules/version_7.0/all.dir/ (accessed on 20 June 2023)) using BWA (Burrows-Wheeler Aligner) [109] (version 0.7.17, Li and Durbin, Cambridge, UK) with default parameters. Only reads with an alignment length greater than 50 were retained for G4 peak calling and downstream analyses. SAMtools (version 1.5, Li, Heng et al., Cambridge, UK, option-markdup) was used to remove PCR duplicates. MACS2 [110] (version 2.1.1, Zhang et al., 2008, MA, USA) was used for the labeling of IP-G4s peaks with the following parameters: macs2 callpeak-g 3.8 × 10^8^-f BAM-extsize-*p* 0.01-nomodel. The Spearman’s rank correlation coefficients between biological replicates under input and PDS/PEG conditions were calculated using the plotCorrelation program of deepTools. We obtained two well-correlated biological replicates for each condition (R = 0.90 and 0.87 for K+PEG/PDS, respectively) (Figure S1; Table S1). The peaks shared by the two biological replicates in both conditions were more than 65% correlated. Biologically reproducible G4 peaks were considered IP-G4 peaks with high confidence, using the intersect command of the bedtools package.

### 4.4. Analyses of GC Content and GC/AT Skews

GC content and GC/AT skew were calculated using the following formulas: GC skew = (G − C)/(G + C); AT skew = (A − T)/(A + T); GC content = (C + G)/(A + T + C + G).

### 4.5. Public Data Analyses

ChIP-seq: We downloaded 14 previously published histone modification data and reanalyzed them following our previously published procedures [7]. Briefly we used mapQ > 20 for unique reads from each data for downstream detection. MACS2 was used to label peaks for each marker by comparing the IP data with the input data. DNase-seq (DNase I sequencing): Using published DNase-seq data, F-seq [111] was utilized with a bandwidth of 200 bp and an FDR (false discovery rate) of less than 0.05. FDR denotes the ratio of the DHS of DNase-seq relative to that of the DHS of the 10 random datasets. DNA-6mA IP-seq (6mA immunoprecipitation sequencing): 6mA IP-seq data were analyzed following published procedures [112], MACS2 was used to label 6mA peaks by comparing the IP data with the input data. DRIP-seq (DNA–RNA immunoprecipitation followed by high-throughput DNA sequencing): data were reanalyzed according to the published protocols [7]. BS-seq (bisulfite sequencing): To examine the level of DNA methylation within the G4 regions, methylated cytosines were counted from the total methylated unique mapping sequence using a double-labeled methylation extraction procedure. DNA methylation levels were calculated using the total number of all (C + T) ≥5 at each position. MNase-seq: MNase-seq data were reanalyzed according to the published protocols [113]. All public data used in this study are listed in Table S2.

### 4.6. In Silico Prediction of Motifs

DNA sequences from the center of a G4 peak spanning ±100 bp were extracted for motif screening. G4-related motifs were identified using MEME-ChIP (http://meme-suite.org/tools/meme-chip (accessed on 13 June 2023)), with parameters chosen as minimum width 5 and maximum width 20. All motif candidates were used to further screen *A. thaliana* to match the potential binding of TFs. The threshold for an enriched region is an *E*-value ≤ 10. Only the top 3 significantly enriched motifs with the highest E-values are listed in the text.

### 4.7. Gene Ontology Enrichment Analyses

All IP-G4^+^ overlapping genes were subject to Gene Ontology (GO) term analyses using online tools in Agrigo v2.0 (http://systemsbiology.cau.edu.cn/agriGOv2/ (accessed on 12 June 2023)) with *O. sativa* annotation.

### 4.8. Normalization of Read Counts

Regions ±1 kb from the upstream and downstream of IP-G4^+^ peaks were partitioned into 50 bp windows, and the G4 peaks were evenly distributed into 20 bins for normalization. The reads for each sliding window were first divided by the window length, and then normalized to the total number of uniquely mappable reads across the rice genome (Mb). For all mapped reads, the individual position in the rice genome was used to determine the midpoint of each sequence segment.

### 4.9. In Silico Identification of PQFSs

Putative G-quadruplex sequences (PQFSs) were identified through comprehensive screening of the entire genome using fastaRegexFinder.py [61]. Briefly, to identify PQFSs, the whole genome sequence was computationally scanned using fastaRegexFinder.py (https://github.com/dariober/bioinformatics-cafe/blob/master/fastaRegexFinder.py (accessed on 10 June 2023)). Subtypes of PQFSs were defined according to loop length and G repeats following the published procedures. PQFSs within IP-G4 peaks were identified using their regular expression. The fold enrichment level for each subtype of PQFSs was calculated by comparing them with random controls, which had the same size distribution as the G4 peaks across the genome (bedtools shuffle command, observed values divided by average values of 1000 randomization).

### 4.10. Circular Dichroism (CD) Assay

DNA oligos from PEG/PDS-specific IP-G4s (Table S3) were denatured at 95 °C for 8 min in PEG/PDS buffer (pure water with 150 mM KCl + 40% PEG/5 μM PDS), and then reassociated by letting the temperature gradually cool down to RT. For CD-spectroscopy, 5 μM of each oligo in both PEG- or PDS-specific buffer were scanned at a wavelength ranging from 220 to 320 nm, with 1 nm bandwidth, 0.5 s response time and 1 mm path length on a Chirascan Spectropolarimeter (Ap-plied Photophysics). Data were buffer-subtracted and normalized to provide molar residue ellipticity values and were finally smoothed for visualization.

## Data Availability

The data generated in this study have been submitted to the NCBI Gene Expression Omnibus (GEO; http://www.ncbi.nlm.nih.gov/geo/, accessed on 28 November 2023) under accession number GSE237294.

## Supplementary Materials

The following supporting information can be downloaded at https://www.mdpi.com/article/10.3390/ijms25010634/s1.
