# Supplementary material for: The Epigenomic Features and Potential Functions of PEG- and PDS-Favorable DNA G-Quadruplexes in Rice
Source: Int J Mol Sci. 2024 Jan 4;25(1):634. doi: 10.3390/ijms25010634 (PMC10779103; doi:10.3390/ijms25010634)
Supplement: Supplementary file 1 [file ijms-25-00634-s001.zip › ijms-2773032-supplementary.pdf]

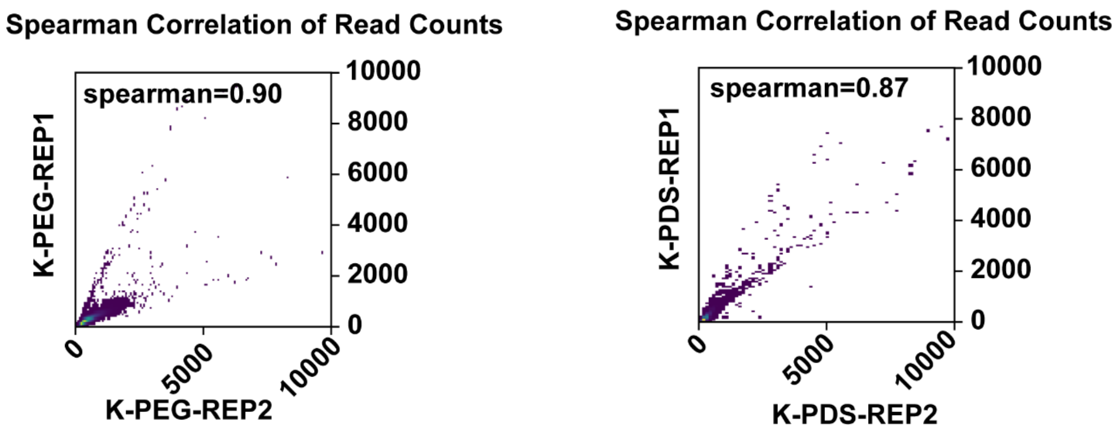

Figure S1. Correlation analyses of biological replicates of BG4-DNA-IP-seq data sets in the presence of PEG/PDS conditions.

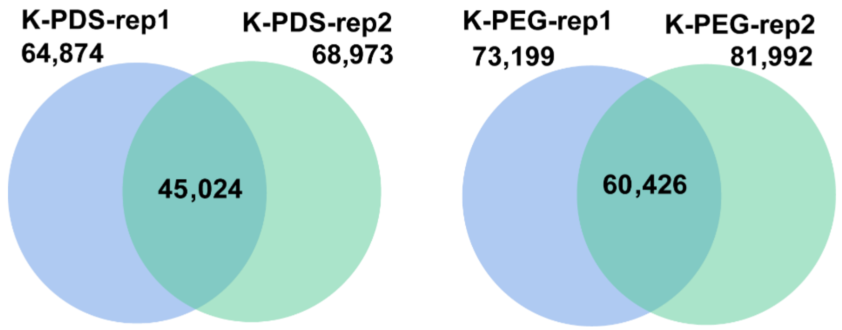

Figure S2. Venn diagrams showing overlaps of IP-G4 peaks between two biological replicates under PEG or PDS conditions, respectively.

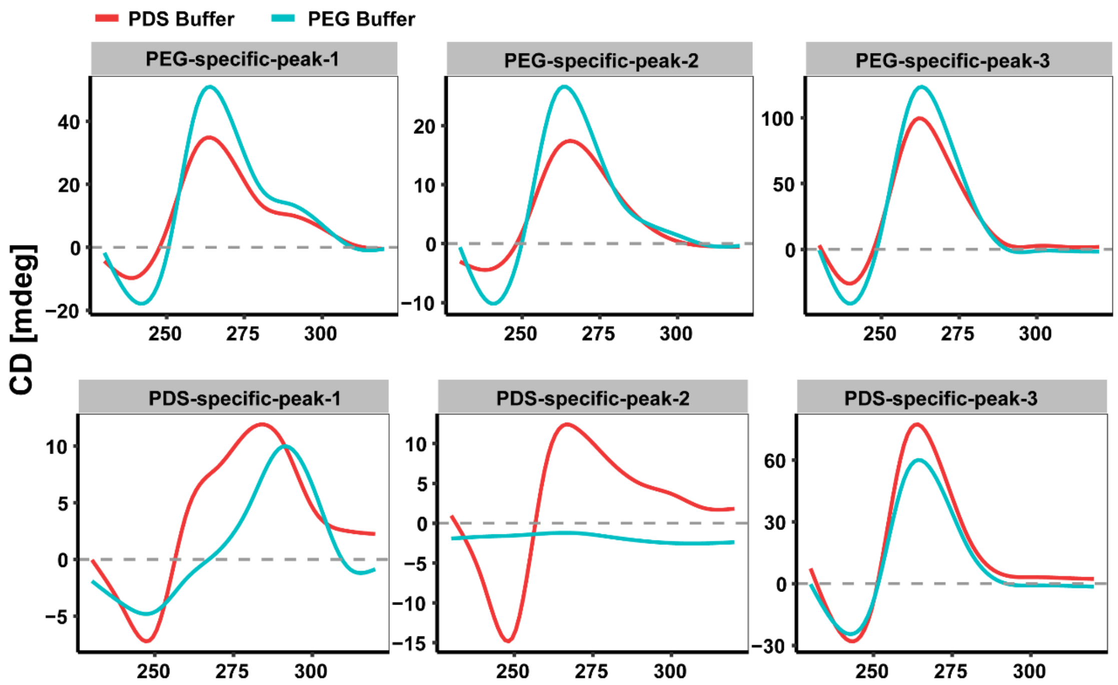

Figure S3. Circular dichroism (CD) spectroscopy assays. Synthesized oligoes corresponding to PEG (top) /PDS (bottom) -specific IP-G4s, reconstructed in both PEG (blue) and PDS (red) specific buffer, respectively for CD assay.

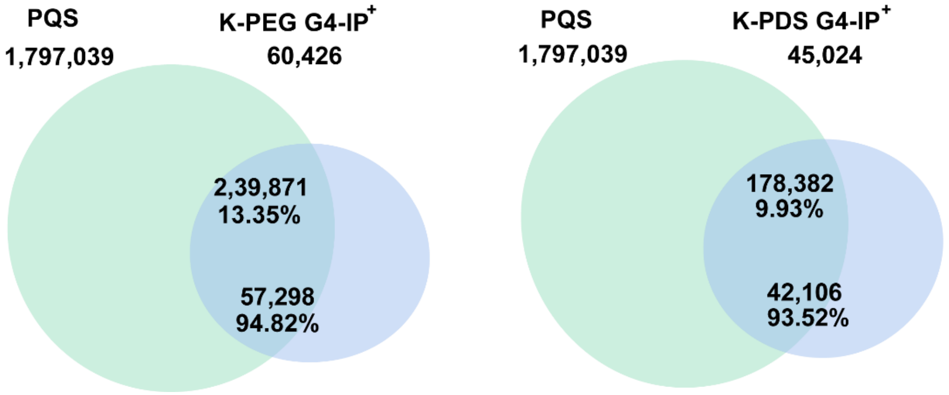

Figure S4. Association analyses of IP-G4s detected under PEG and PDS conditions with predicted PQFSs.

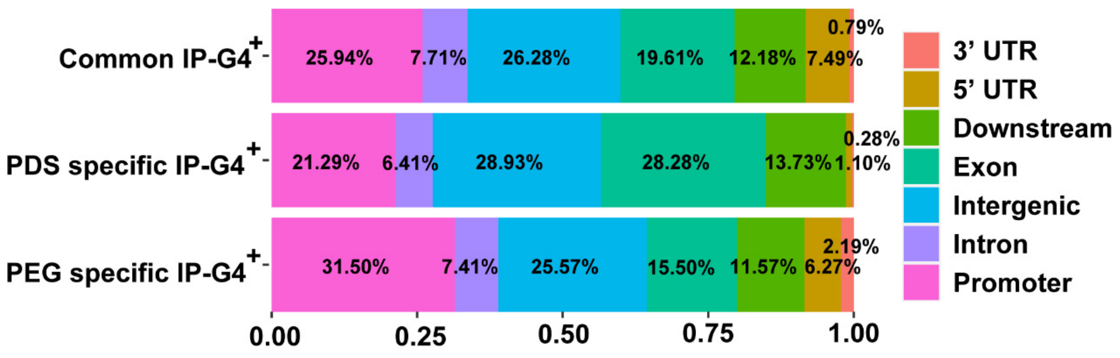

Figure S5 Distributions of three subtypes of IP-G4s peaks (common, PEG/PDS-specific ones) across the rice genome.

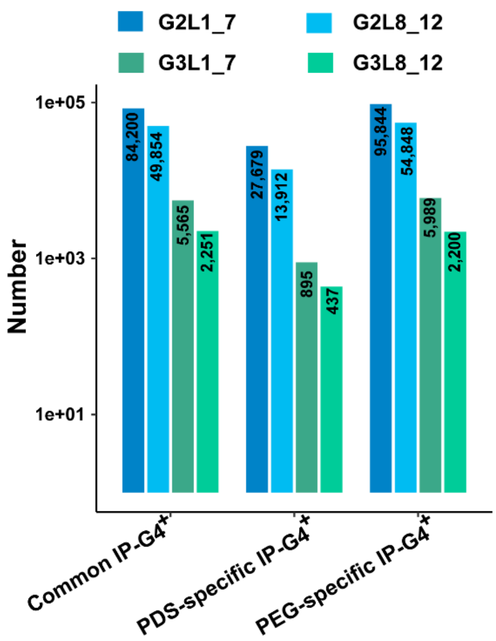

Figure S6. The number of each subtype of PQSs as indicated in PDS-/PEG-specific and common IP-G4s.

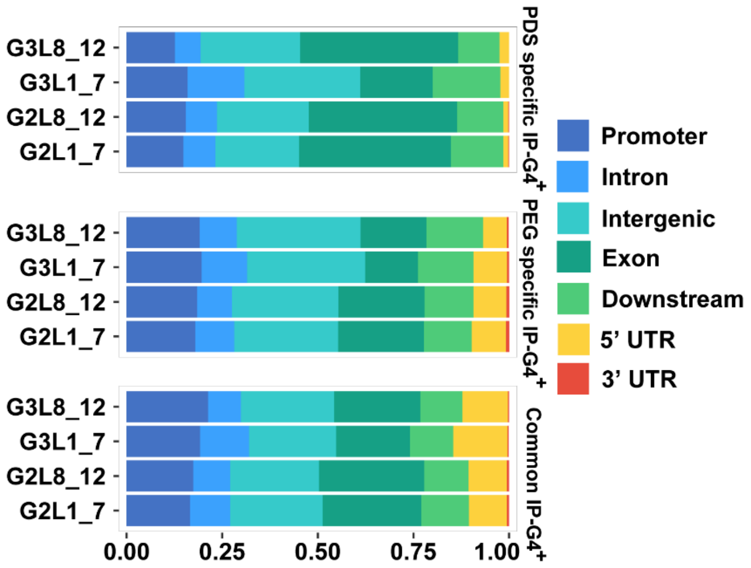

Figure S7. Distributions of subtypes of PQFSs corresponding to PDS-/PEG-specific and common IP-G4s, respectively, in each subgenomic region as indicated.

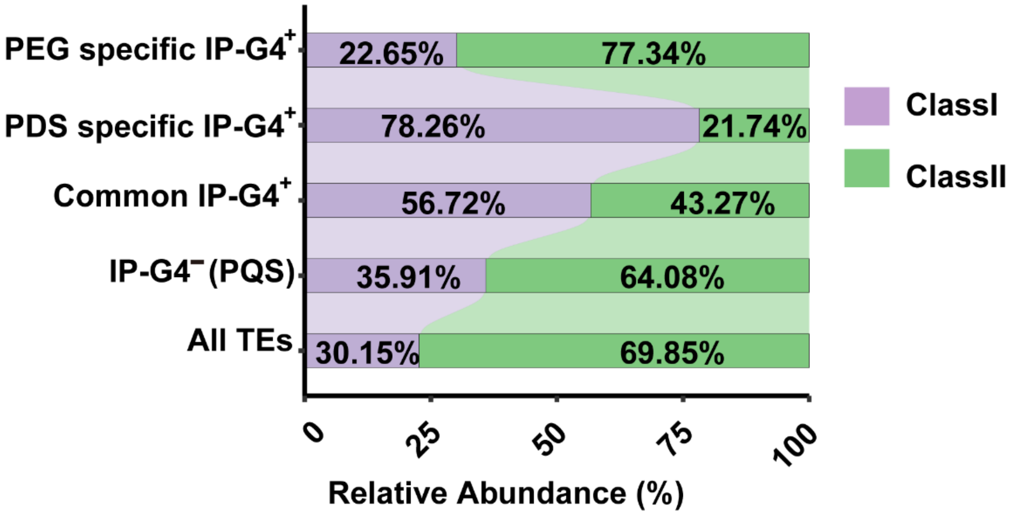

Figure S8. Distributions of PDS-/PEG-specific and common IP-G4^{+} (PQFSs), and IP-G4^{-} (PQFSs) within class I and class II TEs.

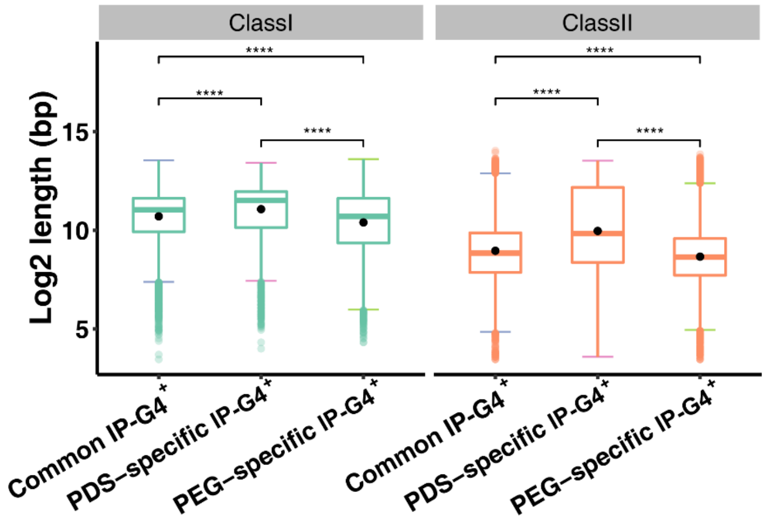

Figure S9. The length of Class I and II TEs associated with each subtype of IP-G4$^+$ as indicated.

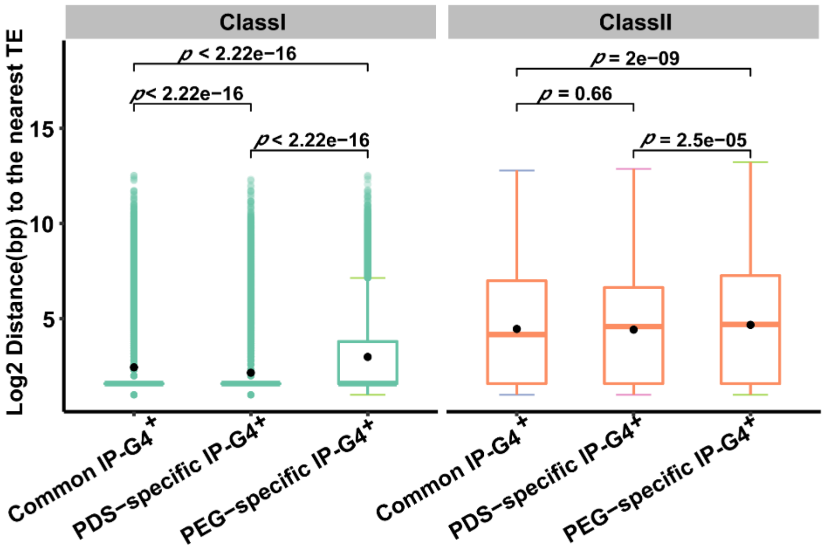

Figure S10 The distance of Class I and II TEs associated with each subtype of IP-G4$^+$ as indicated to the nearest TEs.

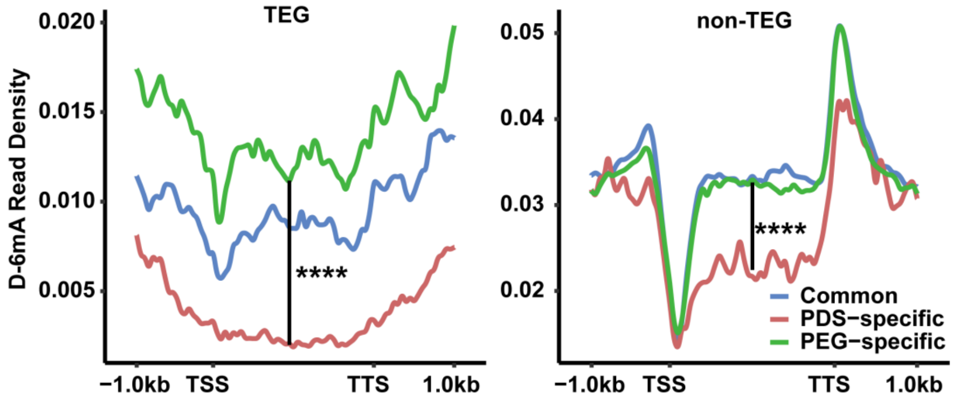

Figure S11. DNA-6mA methylation levels in non-TEGs and TEGs associated with PDS-/PEG-specific and common IP-G4$^+$. Read density of DNA 6mA was plotted across 1 kb upstream of TSSs to 1 kb downstream of TTSs of genes.

Table S1. Summary of sequencing data used in this study.

Samples	Clean reads	Mapping reads	Mapping ratio	Unique reads	Peaks
K-PDS-REP1	146,010,703	64,365,431	44.08%	14,338,375	64,874
K-PDS-REP2	159,213,960	69,531,988	43.67%	17,716,417	68,973
K-PEG-REP1	55,500,244	45,680,377	82.31%	21,134,465	73,199
K-PEG-REP2	80,690,945	71,857,860	89.05%	37,099,992	81,992

Table S2. Summary of published data sets used in this study.

Data	Accession number	References
DNase-seq	GSE26734	Zhang et al. 2012
DRIP-seq	GSE111944	Fang et al. 2019
BS-Seq	SRP238686	Zhao L et al.2020
DNA-6mA IP-seq	GSE103145	Zhou et al. 2018
RNA-seq	GSE33265	Wu et al. 2011
MNase-seq	SRP045236	Zhang et al. 2016
H3K4me3-ChIP-seq	GSE19602	Vaquero-Sedas et al. 2012
H3K36me3-ChIP-seq	GSE26733	Zhang et al. 2012
H3K4me2-ChIP-seq	GSE26733	Zhang et al. 2012
H4K12ac-ChIP-seq	GSE26733	Zhang et al. 2012
H3K9ac-ChIP-seq	GSE79033	Fang et al. 2016
H3K27ac-ChIP-seq	GSE79033	Fang et al. 2016
H3K4ac-ChIP-seq	GSE79033	Fang et al. 2016
H3K9me1-ChIP-seq	GSE79033	Fang et al. 2016
H3K27me3-ChIP-seq	GSE79033	Fang et al. 2016
H3K9me3-ChIP-seq	GSE79033	Fang et al. 2016
H4K16ac-ChIP-seq	GSE69426	Lu et al. 2015
H3K23ac-ChIP-seq	GSE69426	Lu et al. 2015
H3K9me2-ChIP-seq	GSE81436	Tan et al. 2016
H3K4me1-ChIP-seq	SRP238435	Zhao et al. 2020

Table S3. Synthesized DNA oligoes used in this research.

Name	Sequence (5' to 3')
PEG-specific-peak-1	GTAGGGGCGGCGGCGAGGGGCGGCGG
PEG-specific-peak-2	CGAAGGAGGGGGAAGGCCT
PEG-specific-peak-3	AGGCGGGGGGGGGGGGGGGGGCAAG
PDS-specific-peak-1	TGGCACGGCGGCCGGAATG
PDS-specific-peak-2	AGGTTCATATGGAGCTGGTGCTAGAGCCATATAGGC
PDS-specific-peak-3	AGGGGGGTACAAGGGGATAATAATATAGGGGT
